# Supplementary material for: Common Myelin Regulatory Factor Gene Variants Predisposing to Excellence in Sports
Source: Genes (Basel). 2021 Feb 11;12(2):262. doi: 10.3390/genes12020262 (PMC7917663; doi:10.3390/genes12020262)
Supplement: Supplementary file 1 [file genes-12-00262-s001.zip › Table S2.docx]

| SNP | | | | All (athletes+control) | | | | | Control | | | | | Athletes | | | | |
| --- | --- | --- | --- | --- | --- | --- | --- | --- | --- | --- | --- | --- | --- | --- | --- | --- | --- | --- |
| CHR | ID | Minor Allele | Major Allele | MAF | O(HET) | E(HET) | HWE | MISS | MAF | O(HET) | E(HET) | HWE | MISS | MAF | O(HET) | E(HET) | HWE | MISS |
| 11 | rs2286008 | T | G | 0.00037 | 0.001 | 0.001 | 1.000 | 0.0000 | 0.00069 | 0.001 | 0.001 | 1.000 | 0.00000 | 0.00000 | 0.000 | 0.000 | 1.000 | 0.00000 |
| 11 | rs2238001 | C | T | 0.13030 | 0.240 | 0.227 | 0.031 | 0.0000 | 0.12640 | 0.239 | 0.221 | 0.028 | 0.00000 | 0.13470 | 0.241 | 0.233 | 0.497 | 0.00000 |
| 11 | rs198459 | A | G | 0.16700 | 0.276 | 0.278 | 0.697 | 0.0007 | 0.17080 | 0.278 | 0.283 | 0.600 | 0.00138 | 0.16280 | 0.273 | 0.273 | 1.000 | 0.00000 |
| 11 | rs200370195 | - | G | 0.00000 | 0.000 | 0.000 | 1.000 | 0.0000 | 0.00000 | 0.000 | 0.000 | 1.000 | 0.00000 | 0.00000 | 0.000 | 0.000 | 1.000 | 0.00000 |
| 11 | rs139124174 | T | C | 0.00037 | 0.001 | 0.001 | 1.000 | 0.0000 | 0.00069 | 0.001 | 0.001 | 1.000 | 0.00000 | 0.00000 | 0.000 | 0.000 | 1.000 | 0.00000 |
| 11 | rs149803 | G | C | 0.24010 | 0.356 | 0.365 | 0.373 | 0.0000 | 0.24860 | 0.351 | 0.374 | 0.111 | 0.00000 | 0.23050 | 0.361 | 0.355 | 0.738 | 0.00000 |
| 11 | rs139799827 | - | C | 0.00000 | 0.000 | 0.000 | 1.000 | 0.0022 | 0.00000 | 0.000 | 0.000 | 1.000 | 0.00276 | 0.00000 | 0.000 | 0.000 | 1.000 | 0.00156 |
| 11 | rs143144043 | - | C | 0.00000 | 0.000 | 0.000 | 1.000 | 0.0000 | 0.00000 | 0.000 | 0.000 | 1.000 | 0.00000 | 0.00000 | 0.000 | 0.000 | 1.000 | 0.00000 |
| 11 | rs143799782 | T | C | 0.00037 | 0.001 | 0.001 | 1.000 | 0.0000 | 0.00069 | 0.001 | 0.001 | 1.000 | 0.00000 | 0.00000 | 0.000 | 0.000 | 1.000 | 0.00000 |
| 11 | rs146348968 | - | A | 0.00000 | 0.000 | 0.000 | 1.000 | 0.0000 | 0.00000 | 0.000 | 0.000 | 1.000 | 0.00000 | 0.00000 | 0.000 | 0.000 | 1.000 | 0.00000 |
| 11 | rs139188067 | - | C | 0.00000 | 0.000 | 0.000 | 1.000 | 0.0000 | 0.00000 | 0.000 | 0.000 | 1.000 | 0.00000 | 0.00000 | 0.000 | 0.000 | 1.000 | 0.00000 |
| 11 | rs143059056 | T | C | 0.00037 | 0.001 | 0.001 | 1.000 | 0.0000 | 0.00069 | 0.001 | 0.001 | 1.000 | 0.00000 | 0.00000 | 0.000 | 0.000 | 1.000 | 0.00000 |
| 11 | rs174528 | C | T | 0.33600 | 0.454 | 0.446 | 0.545 | 0.0000 | 0.33150 | 0.456 | 0.443 | 0.502 | 0.00000 | 0.34110 | 0.452 | 0.450 | 0.930 | 0.00000 |
| 11 | rs144177087 | G | C | 0.00659 | 0.013 | 0.013 | 1.000 | 0.0000 | 0.00483 | 0.010 | 0.010 | 1.000 | 0.00000 | 0.00857 | 0.017 | 0.017 | 1.000 | 0.00000 |
| 11 | rs141597490 | - | G | 0.00000 | 0.000 | 0.000 | 1.000 | 0.0000 | 0.00000 | 0.000 | 0.000 | 1.000 | 0.00000 | 0.00000 | 0.000 | 0.000 | 1.000 | 0.00000 |
| 11 | rs7943728 | A | G | 0.13480 | 0.221 | 0.233 | 0.063 | 0.0007 | 0.13280 | 0.216 | 0.230 | 0.104 | 0.00138 | 0.13710 | 0.227 | 0.237 | 0.317 | 0.00000 |
| 11 | rs201384645 | A | G | 0.00037 | 0.001 | 0.001 | 1.000 | 0.0000 | 0.00069 | 0.001 | 0.001 | 1.000 | 0.00000 | 0.00000 | 0.000 | 0.000 | 1.000 | 0.00000 |
| 11 | rs61747222 | A | G | 0.03993 | 0.074 | 0.077 | 0.165 | 0.0007 | 0.03936 | 0.073 | 0.076 | 0.303 | 0.00000 | 0.04056 | 0.075 | 0.078 | 0.277 | 0.00156 |
| 11 | rs35113793 | A | G | 0.00110 | 0.002 | 0.002 | 1.000 | 0.0000 | 0.00069 | 0.001 | 0.001 | 1.000 | 0.00000 | 0.00156 | 0.003 | 0.003 | 1.000 | 0.00000 |
| 21 | rs762178 | A | G | 0.44290 | 0.479 | 0.494 | 0.273 | 0.0000 | 0.44480 | 0.489 | 0.494 | 0.821 | 0.00000 | 0.44080 | 0.467 | 0.493 | 0.200 | 0.00000 |
| 22 | rs147817756 | - | G | 0.00000 | 0.000 | 1.000 | 0.000 | 0.0000 | 0.00000 | 0.000 | 0.000 | 1.000 | 0.00000 | 0.00000 | 0.000 | 0.000 | 1.000 | 0.00000 |
| 22 | rs139884 | A | G | 0.37120 | 0.463 | 0.467 | 0.772 | 0.0000 | 0.37430 | 0.470 | 0.468 | 1.000 | 0.00000 | 0.36760 | 0.455 | 0.465 | 0.611 | 0.00000 |
| 22 | rs147334218 | A | G | 0.00293 | 0.006 | 0.006 | 1.000 | 0.0007 | 0.00415 | 0.008 | 0.008 | 1.000 | 0.00138 | 0.00156 | 0.003 | 0.003 | 1.000 | 0.00000 |
| 22 | rs138500876 | - | C | 0.00000 | 0.000 | 0.000 | 1.000 | 0.0000 | 0.00000 | 0.000 | 0.000 | 1.000 | 0.00000 | 0.00000 | 0.000 | 0.000 | 1.000 | 0.00000 |
| 22 | rs149435516 | A | G | 0.05040 | 0.092 | 0.096 | 0.150 | 0.0051 | 0.05556 | 0.103 | 0.105 | 0.477 | 0.00553 | 0.04460 | 0.080 | 0.085 | 0.124 | 0.00467 |
| 22 | rs148688873 | C | A | 0.02672 | 0.053 | 0.052 | 0.622 | 0.0000 | 0.02831 | 0.057 | 0.055 | 1.000 | 0.00000 | 0.02492 | 0.050 | 0.049 | 1.000 | 0.00000 |
| All | | Mean | | 0.07507 | 0.105 | 0.145 | 0.719 | 0.0004 | 0.07560 | 0.106 | 0.107 | 0.806 | 0.00048 | 0.07448 | 0.104 | 0.105 | 0.834 | 0.00030 |
|  |  | SD | | 0.13052 | 0.166 | 0.241 | 0.379 | 0.0011 | 0.13104 | 0.167 | 0.168 | 0.332 | 0.00123 | 0.13000 | 0.165 | 0.167 | 0.294 | 0.00099 |

CHR-chromosome; MAF – minor allele frequency; O(HET) – observed heterozygosity; E(HET) – expected heterozygosity; HWE – exact test p-value for HWE; MISS – percentage of missing genotypes
